# Healthcare providers’ perspectives on antimicrobial resistance in Northwest Syria: an exploratory qualitative study

**DOI:** 10.3389/fpubh.2025.1662934

**Published:** 2025-12-03

**Authors:** Hussam Alkabbani, Maysoon Dahab, Waseem Zakaria, Ahmad Hmeideh, Nasser Almhawish, Nabil Karah, Aula Abbara

**Affiliations:** 1 Department of Infectious Diseases Epidemiology and International Health, London School of Tropical Medicine and Hygiene, London, United Kingdom; 2 Department of Medicine, University of Aleppo, Aleppo, Syria; 3 Syria Public Health Network, London, United Kingdom; 4 Department of Molecular Epidemiology, University of Umea, Umeå, Sweden; 5 Department of Infectious Diseases, Imperial College, London, United Kingdom

**Keywords:** Syria, AMR, antimicrobial resistance, antimicrobial stewardship, conflict

## Abstract

**Background:**

Armed conflict contributes to the development and spread of antimicrobial resistance (AMR). Understanding healthcare providers’ perceptions is essential for identifying key determinants and developing contextualised recommendations. This study explored perceptions of AMR among healthcare providers in northwest Syria - a conflict-affected area- before the fall of the Syrian regime in December 2024.

**Methods:**

A qualitative study was conducted using semi-structured interviews with healthcare providers who had worked in northwest Syria within the previous five years. Seventeen interviews were conducted remotely in Arabic in July–August 2023. Participants were identified through purposive and snowball sampling. Transcriptions were analysed using deductive thematic analysis.

**Results:**

Of the 17 key informants, (11 doctors, 6 pharmacists); most were from Idlib governorate. Emerging themes included healthcare professionals’ understanding of AMR, perceived drivers, conflict- and disaster-related influences, and examples of good practice. Frequently cited drivers of AMR were over-the-counter availability of antimicrobials, pharmacy-led dispensing, and community insistence on antibiotics. Inadequate microbiology diagnostic capacity was also highlighted as a key factor.

**Conclusion:**

Interviews highlighted perceived contributors to AMR in northwest Syria, including ineffective or absent policies regulating antibiotic dispensing, lack of antimicrobial guidelines or poor enforcement, and limited access to diagnostics. These findings support the development of contextualised recommendations during the transition of Syria’s health system.

## 1 Introduction

Protracted armed conflict contributes to the development of antimicrobial resistance (AMR) through multiple pathways including the breakdown of healthcare infrastructure, increase burden of traumatic injuries and disruption of governance and regulatory measures (1). Conflict also undermines healthcare professionals’ capacity through disrupted training and the loss of experienced staff (2). Data on AMR in conflict affected settings suggests high rates of multidrug resistant organisms (MDROs,) particularly for Gram negatives including ESBLs (Extended Spectrum beta-lactamases) and CREs (Carbapenem Resistant Enterobacteriaceae) (3). Data from Iraq (4), Yemen (5), Ukraine (6), Syria (7) and Gaza (8) suggest high rates of MDROs in these conflict zones.

In Syria, robust data on AMR are sparse both before and after the onset of armed conflict in March 2011 (7). Additionally, the impact of the conflict on Syria’s health system has been profound and has provided an ecosystem in which AMR can develop and spread. Over the duration of the conflict, Syria’s health system was fragmented into at least four regions of control including northwest, northeast, former regime-controlled areas and areas formerly under Turkish control. Among other impacts, this also negatively impacted governance around AMR, particularly over the counter antibiotics without prescription. The extent of this has only fully become apparent after the fall of the regime in December 2024, where the extent of health system under-resourcing has become more evident in areas formerly under regime control.

Abbara *et al* describe the limited available literature on AMR in Syria and the range of pre and post conflict exacerbating drivers (7). However, data on MDROs since the onset of the conflict are sparse and of limited quality (3, 7). Additionally, areas outside of former regime control, including former northwest Syria were excluded from AMR planning by the former regime. For example, although the former Syrian Ministry of Health launched a National Action Plan on AMR in 2019, its implementation focused only on areas under its control at that time, excluding northwest Syria; even in those areas, implementation was limited (9).

For former northwest Syria, there were numerous challenges to the health system which affected the identification and response to MDROs. These include limited access to diagnostics, poor governance for quality control of existing microbiology laboratories (many of which were private,) and poor practices related to overuse and misuse of antibiotics and poor-quality antibiotics (10–12). In addition, the February 2023 earthquakes also added additional challenges for AMR related to an increase in injuries (including severe and crush injuries), damage to infrastructure and misuse of antibiotics.

Though there were some efforts to support diagnostics and improve antimicrobial stewardship in the former northwest Syria, these were limited and not always effective. One example is that in early 2024, the WHO-led Health Cluster, based in Gaziantep (Turkey) introduced antimicrobial protocols for primary care, alongside target training for healthcare staff through the different humanitarian organizations active in that region (13).

The aim of this study was to explore the perceptions of Syrian healthcare providers (physicians and pharmacists,) on the drivers of AMR in the former northwest Syria, the impact of conflict and earthquakes and what examples of good practice existed at the time of the interviews. This will support contextually relevant recommendations.

## 2 Materials and methods

We used a qualitative study methodology to gain an in-depth understanding of perceptions of AMR among healthcare professionals in northwest Syria. Given the security situation at the time of the interviews (July–August 2023,) interviews were conducted remotely, an approach which has been used successfully in other qualitative research in the geographical area (14, 15).

### 2.1 Study setting

At the time of the study, northwest Syria was an area of around 5.1 million people, of whom more than two thirds were internally displaced people (IDPs) with around 2 million living in tented settlements, a number which increased after the February 2023 earthquakes (16). It described most of Idlib governorate, and parts of Aleppo governorate. The Syrian Ministry of Health had withdrawn from this area soon after the uprisings leading Syrian and international humanitarian organizations to step into the health space; in 2013, Idlib Health Directorate was established as an independent locally elected health authority to fill the void left by the exodus of the Syrian Ministry of Health (17). The health system had been severely weakened by the protracted conflict and the increasing health needs of the population alongside severe under-resourcing (18). There was also a growing, under-explored, and poorly regulated private health system (19).

### 2.2 Participants and sampling approach

In this exploratory, qualitative research, we focused on the perspectives of doctors and pharmacists, the two main groups involved in prescribing and dispensing antibiotics in the former northwest of Syria. We used a combination of purposive and snowball sampling to identify physicians and pharmacists who fulfilled the inclusion criteria (see Table 1). Initial participants were identified through the Syrian Board of Medical Specialties (SBOMS) and Syria Public Health Network (SPHN) and the contacts of authors. Specifically, we shared information about the study in a WhatsApp group with senior SBOMS staff, who then nominated the first group of participants from their network, who met the inclusion criteria.

**Table 1:** This table summarize the study participants’ Inclusion Criteria.

**Inclusion criteria**
Physicians or pharmacists currently practising or had been practising in northwest Syria in the previous 5 years
Manage patients who require antimicrobials as part of their clinical work or are involved in the clinical management of infectious diseases.
Aged 18 years and over
Are willing and able to meet virtually
Able to consent
Able to speak Arabic

After the initial interviews, we undertook snowball sampling by asking the initial participants to nominate other participants who also met the inclusion criteria. We actively sought to interview pharmacists and female participants as these groups were often under-represented in similar research. Participants who were unwilling to consent and did not fulfil the inclusion criteria were excluded.

On identifying potential participants, an invitation letter was sent in Arabic for them to read and consider. Healthcare professionals who gave their initial permissions were then contacted and provided with the Participant Information Sheet (PIS) written in Arabic. All participants were given 72 h to read the PIS and confirm participation and were contacted to answer any questions or clarifications at this stage. When the participants confirm their participation, an informed consent form was sent electronically to be signed before proceeding with the interview. In some cases, obtaining electronic consent was challenging, so consent was recorded verbally instead.

Upon receiving the consent from the participants, the interviews were scheduled. All interviews were conducted remotely using WhatsApp application, which is commonly used in the region, and has end-to-end encryption. All interviews were conducted in Arabic (Syrian dialect), which is the native language for both the participants and the main researcher HQ. All interviews were recorded after the approval from the participants and were transcribed verbatim through an Sonix.ai application in Arabic. All transcriptions were then reviewed and corrected in order to be ready for analysis. The interviews were all conducted between 12/07/2023 and 5/8/2023; all were between 30 and 45 min, except for two interviews which lasted for around 60–70 min.

### 2.3 Data collection tool

An interview guide was developed with semi-structured open questions; the guide was written in English then translated to Arabic. The guide had sets of questions under three themes of (1) Knowledge, Attitude, Practices (KAP) on extent, drivers, guidelines and management of AMR. (2) Perceived effect of the armed conflict and earthquake on AMR (3) Examples of good practice or recommendations on addressing AMR in northwest Syria. The interview guide is available as Appendix.

### 2.4 Data analysis

After data transcription through Sonix.ai, and manual validation, the data were analyzed using qualitative content analysis using Excel, adopting an inductive approach to explore the unique context, and to understand the complexity of the situation with open approach to new insights. The data were then coded, and distinct labels were assigned to relevant segments. This process paved the way for the formation of categories and themes of analysis. Subsequently, a thematic framework was constructed, organizing the identified topics through indexing, sorting, assigning symbols, and establishing connections among them. Findings were discussed among authors during the data analysis phase to come to a mutual understanding regarding the emerging themes and codes. All codes and themes were identified in Arabic first, then translated into English. This is because the researcher is fluent in Arabic, can understand the culture and language nuances, allowing a more robust, richer analysis to take place.

### 2.5 Partnerships

To ensure the relevance of this work to the local health system and the local healthcare professionals, we engaged early with local health actors including SBOMS who gave direct feedback on the aims of the project and the questionnaires. SBOMS is a Syrian independent academic body that awards medical specialization to resident doctors in northwest Syria. We also engaged with SPHN, a UK based organization which has conducted research and policy on Syria since 2015 and R4HSSS (Research for Health Systems Strengthening in Northern Syrian), an NIHR funded research program based at King’s College, London. They supported with feedback and advice about situating the findings of the research in the wider research on the health system in northern Syria.

### 2.6 Ethical considerations

Ethical approval for this study was obtained from the Ethical Review Committee at the London School of Hygiene and Tropical Medicine “LSHTM,” with the reference number: 29400/RR/32188. While obtaining local ethical approval was not feasible due to the absence of an ethical committee in the region, permission to conduct the research was granted by the Idlib Health Directorate, where the participants were based. Throughout the study, no distressing or uncomfortable emotions were observed during the interviews, as they were conducted as scientific discussions. To protect the confidentiality of data and the privacy of participants, all data were anonymized and assigned a random number with only the main researcher having access to the coding guide. All the data were stored in a single password protected Sonix account, and a single password protected drive. Key informants were informed that all data will be destroyed 5 years after the research is completed and published. Key informants were aware that they could withdraw their participation at any time and all information relevant to the participant would be deleted.

## 3 Results

A total of 21 participants were approached; two declined to participate and two were unable to schedule an interview during the data collection period; as such 17 interviews were conducted. The interviews were transcribed and analyzed alongside data collection, enabling an iterative process of coding and theme identification. Data saturation was reached by the time the 17 interviews were conducted, as no new concepts emerged in subsequent interviews. The recurring reinforcement of themes confirmed the comprehensiveness of our data and justified concluding the sampling process.

Of the 17, 12 were male and five were female. Eleven were physicians and six were pharmacists; 15 of them were practicing in Idlib governorate at the time of the interview. Table 2 has more details of the participants.

**Table 2:** This table lists the participants, their age range, location, and their medical specialty:

Key informants characteristics	*n* (%)
Age	
*22–29*	3 (18)
30–39	8 (47)
40–49	6 (35)
Gender	
Male	12 (71)
Female	5 (29)
Place of Practice	
Idlib governorate	15 (88)
Aleppo governorate	2 (12)
Speciality	
Internal Medicine with Intensive Care	3 (17)
Neonatal Intensive Care Resident	2 (12)
General Paediatrician	2 (12)
Internal Medicine	2 (12)
Orthopaedic surgeon	1 (6)
Nephrologist	1 (6)
Pharmacist	6 (35)

**Figure 1:**
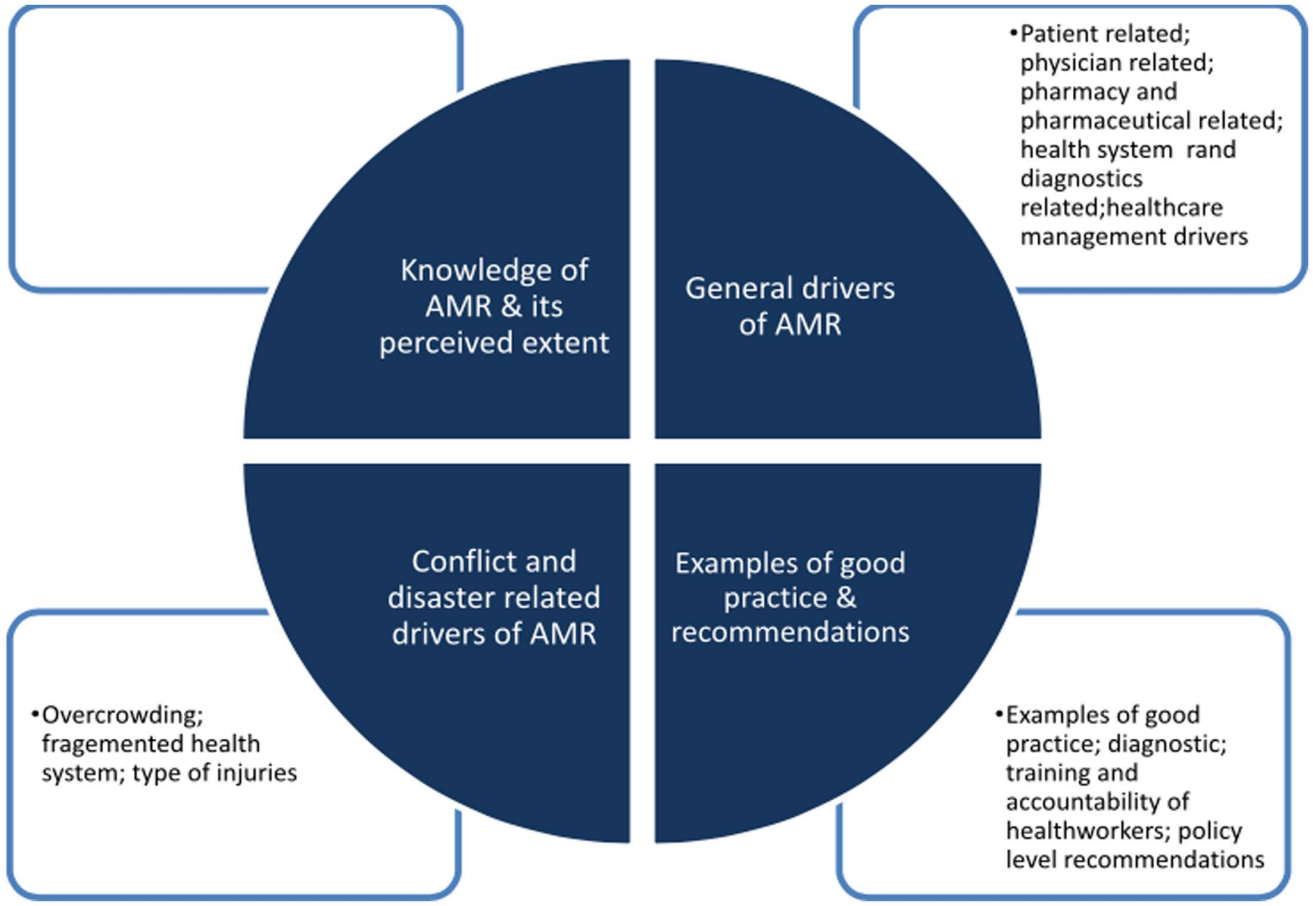
The main emerging themes (inner circle) and sub-themes (squares) are summarised in this figure.

The main findings are categorized in four main themes, three of which have sub-themes; these are summarized in Figure 1. The main themes were pre-set through the study objectives, while the sub-themes were listed according to priorities and frequency of mention by the participants.

### 3.1 Knowledge of AMR and its perceived extent

All participants had an overall good understanding of AMR, in line with the WHO definition (20). However, none were familiar with the term antimicrobial stewardship (AMS) and how it could be implemented in clinical practice though they were aware of some the concepts on direct questioning. Participants’ perspectives were that AMR was widespread in northwest Syria citing examples of antibiotic failure. However, they also noted that for much of their clinical practice, they did not have diagnostics to support this assumption due to poor availability and quality of diagnostics. As such, they acknowledged that this assumption was based on a lack of response to commonly used antibiotics. A paediatrician described concerns about antimicrobial resistance stating: “… despite the overall lack of culture testing, when we … conduct urine cultures, most of the results show resistance to multiple antibiotics, which is usually effective on the bacteria that cause urine infections.”

Where cultures were available, particularly in intensive care, participants gave examples such as *Pseudomonas aeruginosa*, a Gram-negative rod which has intrinsic resistance to many commonly used first line antibiotics. A neonatal intensive care specialist said: “*Pseudomonas aeruginosa* is found in many hospitals in the northwest of Syria; it goes when they close the ward and disinfect it, but it soon comes back again.”

All participants reported that understanding and tackling AMR is important now, even those who did not know think that the current prevalence is high. One participant described the situation as “catastrophic” and multiple participants reported cases where urine culture showed resistance to all antibiotics tested or available in the area. Some participants reported that they were concerned that this contributed to the use of second- or third line antibiotics and possibly led to harm. A paediatrician noted his concerns stating: “Sometimes I feel very embarrassed with the patients. Even if a child is an outpatient, if the parents bring me a urine culture result which is only sensitive to meropenem for example, or the second and third lines of treatment, which has no oral formula, I must admit the child to hospital for only a simple urine infection.”

### 3.2 General drivers of AMR

Participants described a range of drivers of AMR and reported that addressing all would be challenging. “We have a treatment chaos; it’s a circle started from pharmacists to patients and ended with physicians.” Drawing on the drivers reported, we classified the drivers into five sub-themes as follows: (1) Patient related. (2) Physician related. (3) Pharmacy and pharmaceutical related. (4) Health system and diagnostics related (5) Healthcare management drivers. The sub-themes were prioritized according to the frequency of mention by the participants.

#### 3.2.1 Patient related drivers

Patients’ non-compliance to antibiotics and self-medication were the most described, perceived driver of AMR, by the participants. Noncompliance could be by taking the medication less or more frequently, not completing the treatment course, or using different doses. This is reported to be due to a lack of awareness of the impact or sometimes due to financial constraints. A pharmacist stated: “Patients will come and ask for half of the prescribed course, because they are trying to reduce the costs.”

Self-medication without a prescription is widespread. A participant noted, “It happens many times that when I prescribe Augmentin [co-amoxiclav] for a sick child and check on the patient 2–3 days later, the mother says, ‘The medication is finished.’ When I inquire why it’s finished so quickly, she responds, ‘His siblings were also sick, so I gave them the same medicine.’”

Patients’ financial considerations were also reported to have an impact. For example, they may not be able to pay for microbiology testing and may go straight to a pharmacy directly rather than the overcrowded public health system or expensive private health facilities. A physician stated, “Only one in 10 patients are able to pay for culture sensitivity tests; this number decreases to one in a hundred in camps.”

#### 3.2.2 Physicians related drivers

##### 3.2.2.1 The necessity and chaos of empirical treatment

The primary driver cited was the widespread reliance on empirical antibiotic treatment. Empirical treatment with antibiotics was described as a common practice in northwest Syria. Participants explained that this practice was a direct result of the absence of diagnostic infrastructure, particularly the lack of accessible microbiology laboratories. Participants reported that this systemic barrier forces physicians to rely on clinical judgment and best-guess estimates, leading to high variability and a lack of monitoring in prescribing practices.

A physician described this practice as “empirical treatment is fundamental in our daily practice, but its implementation is often chaotic. There are distinct guidelines for empirical treatment that not all physicians are aware of. For instance, while an NGO (non-governmental organization) warehouse might be full of amoxicillin, azithromycin will always be depleted. Sometimes, a case demands a precise treatment, but in the absence of sensitivity tests, empirical treatment is our only option, so some patients may deteriorate under this approach, potentially progressing to septic shock.”

##### 3.2.2.2 Irrational prescription of antibiotics

Participants described the irrational prescription of antibiotics on multiple levels, which include, the incorrect combination of antibiotics, prescription without a clear indication, the practice of “aggressive treatment” as a response to expected AMR, and incorrect prescriptions. Furthermore, the analysis revealed a connection between prescribing and patient-related factors. Physician’s fear of damaging their reputation influenced their irrational decision to use antibiotics. A neonatal intensive care specialist said: “I recently saw a patient with a prescription of Augmentin, ceftriaxone, gentamicin, flagyl [metronidazole] and azithromycin, prescribed together!”

#### 3.2.3 Pharmacies/pharmaceutical related drivers

Participants reported that limited regulation about antibiotic prescribing in northwest Syria has transformed pharmacies from points of controlled access into primary, unregulated sources of antibiotics. This role is characterized by three interconnected issues: profit-driven and patient-led dispensing practices, the circulation of substandard antibiotics, and a lack of oversight, all of which are direct consequences of the systemic regulatory vacuum.

##### 3.2.3.1 Unregulated dispensing: profit and patient pressure

In the absence of laws prohibiting the sale of antibiotics without a prescription, financial incentives and patient demand become the primary drivers of dispensing. Pharmacists acknowledged that antibiotics are significant profit-makers, especially with promotional offers from suppliers. This commercial reality conflicts with professional judgment, as pharmacists face direct pressure from patients who will simply seek antibiotics elsewhere if refused. As one pharmacist stated, “To be honest, we open the pharmacy to make profit, and the drug companies are giving great offers on antibiotics.” Another confirmed this pressure, noting, “I tell my patients not to take antibiotics without a need, but they said thanks for the advice but please give me what I want, or I’ll get it from another pharmacy.” This environment fosters practices like the dispensing of a triple injection of “ceftriaxone, dexamethasone, and sodium diclofenac,” which is provided on demand for rapid symptomatic relief without any medical indication.

##### 3.2.3.2 Proliferation of substandard antibiotics

Regulatory failure extends to quality control, allowing substandard or falsified antibiotics to enter the market. With Idlib Health Directorate able to oversee only one official border crossing, antibiotics arriving through other channels may be identical in appearance but less effective or have a shorter shelf life. This issue directly undermines clinical efforts and complicates the perception of resistance. A physician explained this dilemma: “When I am concerned about resistance, the first thing I do is to check what kind of medication the patient is taking, and I have created a blacklist of brands of medications that are not really effective.” Thus, treatment failure may be misattributed to AMR when the root cause is actually a lack of active ingredient in the drug itself.

##### 3.2.3.3 The consequence: a cycle of misuse and AMR

Together, these factors create a self-perpetuating cycle of antibiotic misuse. The easy availability of antibiotics without a prescription fuels patient demand and self-medication. The circulation of substandard drugs leads to treatment failures, which in turn prompts both patients to seek stronger or combination therapies and clinicians to prescribe more aggressively, further accelerating the development of AMR. The pharmacy, in this unregulated context may act as a critical amplifier of the crisis.

#### 3.2.4 Health system and diagnostics

The physician and pharmacy-related drivers of AMR are not isolated issues but are symptoms of a profoundly weakened health system in northwest Syria. The collapse of core regulatory, diagnostic, and service delivery functions has created an environment where irrational antibiotic use is not just common but is often the only feasible option. The systemic failures can be categorized into three critical gaps: governance, diagnostic capacity, and appropriate care models.

##### 3.2.4.1 The governance gap: absence of enforcement and oversight

The foundational driver enabling AMR is the lack of a legally enforced framework to control antibiotics. While regulations exist for other drug classes, the absence of such a prohibition for antibiotics, or a complete lack of enforcement, has normalized their over-the-counter status for decades. A pharmacist’s statement, “The ideal solution is to have a law which prohibits dispensing the medication without prescription, like that which exists for mental health and anaesthesia drugs now,” highlights that the issue is not feasibility but political and institutional will. This regulatory vacuum, as detailed in the pharmacy section, was suggested by participants to be the reason why antibiotics are treated as a market commodity rather than a public health resource, embedding misuse into the very fabric of healthcare access.

##### 3.2.4.2 Operational failure: the diagnostic void and its clinical consequences

The regulatory collapse directly enables an operational failure: The near-total absence of affordable, reliable diagnostic capabilities forces healthcare providers into a cycle of guesswork. Public hospitals lack basic microbiology services, and while limited tests like urine culture are available privately, they are often considered untrustworthy due to poor quality control. A nephrologist’s experience is illustrative: “I had many cases, where the results did not just give that there’s no resistance, but surprisingly it says there’s no bacterial growth!” This diagnostic black hole is the direct cause of the “chaotic empiric treatment” described by physicians. Without accurate data to guide therapy, antibiotics are prescribed blindly, inevitably leading to incorrect choices and driving resistance, while also making surveillance of AMR trends impossible.

##### 3.2.4.3 The service-delivery gap: poorly regulated humanitarian response

Even well-intentioned humanitarian aid models can inadvertently contribute to AMR when designed without addressing its underlying drivers. Mobile Medical Units (MMUs), which are essential for serving displaced populations in most camps, often operate under intense pressure to treat large numbers of patients rapidly, with limited emphasis on quality of care. In some NGOs, leadership had prioritized quantity over quality, structurally incentivizing prescribing practices known to be harmful. One participant illustrated this challenge: “In a mobile unit, I’ll go let us say, with 10 boxes of Augmentin, 10 blister packs of ibuprofen and 10 of ciprofloxacin; for me my job is to distribute them and come back home. For anyone presenting with urinary burning sensation, I’ll give him ciprofloxacin without lab tests or without anything,” highlighting how operational constraints and the absence of technical monitoring in humanitarian responses can institutionalize antibiotic misuse.

#### 3.2.5 Healthcare management

Impaired implementation of Infection Prevention Control (IPC) policies in hospitals in northwest Syria is highlighted by participants as an important contribution to AMR. Although there’s growing interest in IPC policies and programs in the region, the implementation and monitoring of IPC is reportedly inadequate. The current approach of employing small non-medical buildings to be used as hospitals, to maximize protection from direct targeting led to reduced isolation capabilities and an insufficient ventilation system. This approach could potentially increase susceptibility to infections. A paediatrician said: “IPC is approved by NGOs, and there is a lot of training on it, but it’s not implemented, I believe 60–70% of health facilities do not implement or monitor IPC.”

### 3.3 Conflict and natural disaster related exacerbators

#### 3.3.1 Overcrowding as an amplifier of AMR

Beyond the direct drivers of AMR within the health system, the living conditions shaped by protracted conflict and the 2023 earthquake amplified the problem. Overcrowding in IDP camps created a distinct epidemiological environment that could accelerate the development and spread of AMR through three interconnected pathways: heightened infection transmission, compromised antibiotic treatment courses, and environmental contamination.

##### 3.3.1.1 Creating a reservoir for pathogen transmission

The dense living conditions in tented settlements altered disease dynamics. As an internist described, “I mean, regardless of if it’s a viral or bacterial infection, instead of having one unwell patient in a family, the whole family becomes unwell*.”* This high infection rate could increase the community-wide volume of antibiotic use, providing more opportunities for resistance to emerge.

##### 3.3.1.2 Intensified pre-existing financial pressures by compromising treatment adherence

Overcrowding exacerbated the financial precarity discussed earlier, creating a link between living conditions and irrational antibiotic use. The financial strain of treating multiple sick family members simultaneously forced difficult choices. Patients were pushed toward shorter, cheaper treatment pathways, such as not completing a prescribed course or bypassing a physician, creating a vicious cycle where overcrowding leads to misuse, which in turn fuels AMR. An internist said: “Having whole family becomes unwell, will increase the financial burden on the family and can lead to shortening of the treatment pathway to save some costs.”

##### 3.3.1.3 Environmental contamination and bio-burden

Poor sanitation infrastructure, one participant said, “Whole camps having issues with their sewage systems; we hear about camps which had an open sewage drainage.” This contaminated environment not only increased the incidence of infections but also creates a persistent external bioburden. The community exposure to this high bacterial load, including resistant strains, could drive a cycle of recurrent infections and the environmental spread of resistance.

#### 3.3.2 Fragmented health system

Participants described the lack of governance and leadership in the health system in former northwest Syria, which manifested as poor monitoring and supervision of hospitals and health facilities, and a lack of enforced regulations and policies to organize the health system. A pharmacist stated, “There’s no integrated system like before, in order to control the medicines; you need a complete circle and you need multiple efforts to control the border, the market, pharmacies *etc*.” Participants also described the financing of the health system in northwest Syria as a contributing factor to AMR due to the instability of funding streams which impacted the sustainability of programs and medication supplies.

#### 3.3.3 Type of injuries

The nature of conflict and earthquake injuries created a high-risk environment for the emergence of AMR, distinct from routine clinical practice even in low resource settings. These injuries drive antibiotic overuse and AMR through a cascade of clinical challenges, from initial empiric therapy to complex wound management.

##### 3.3.3.1 Empiric overuse in mass casualties

During periods of mass casualties, clinicians frequently resorted to broad-spectrum antibiotics as prophylaxis as a substitute for inadequate IPC or surgery. As one pharmacist recalled, *“When we were accepting mass casualties. The first thing we did for almost every injury was to start ceftriaxone. We were worried. Because the sterilisation. Was not optimal.”* This approach represents a rational clinical response within a collapsed health system, where antibiotics became a compensatory measure for the absence of sterile surgical conditions and the breakdown of IPC infrastructure.

##### 3.3.3.2 Complex wounds and resistant infections

The injuries themselves emerged as a primary driver of resistant infections. War and crush injuries were reported to be deeply contaminated, involved necrotic tissue, or contained retained foreign bodies, creating a biological environment that often required prolonged and escalating antimicrobial regimens. As one orthopaedic surgeon noted, “The presence of a war injury by itself increases the infection rate considerably.” Similarly, the severity and contamination of earthquake-related wounds, combined with delays in seeking care due to access barriers, often prompted the immediate use of broad-spectrum, last-resort antibiotics such as piperacillin–tazobactam and carbapenems. A physician recalled, “We changed the treatment course multiple times, and some patients acquired additional infections in hospital and required longer periods of hospitalization.” Such patterns, in which complex wounds and initial treatment failure drive repeated escalation, directly contribute to the selection of resistant pathogens and the gradual exhaustion of effective therapeutic options.

### 3.4 Current good practice and recommendations

#### 3.4.1 Examples of good practice

The majority of participants strongly emphasized the absence of any coherent and systematic best practices throughout the former northwestern of Syria with available projects, often small scale or unstained. They described a lack of attention from humanitarian organizations, the health directorate or health facilities in addressing AMR. They did note an agenda to improve IPC, mostly pushed by WHO led health cluster in Gaziantep, Turkey which prompted NGOs to implement training and policies; however, this was not always well implemented, sustained or monitored. A pharmacist holding a dual role as a hospital manager shared, “Based on my experience and understanding, aside from IPC, there is a notable lack of systematic practices. While sporadic lectures may occur, there’s an absence of a formalized approach, both from the Health Directorate and NGOs.”

Some other, small-scale examples of good practice were noted; however, they were fragmented. One international humanitarian organization used the WHO AWaRe (Access, Watch, Reserve) system, strictly restricting the use of antibiotics to their protocols. Some participants noted the efforts of SBOMS, through some of its residency programs, to systematically combat the irrational prescription of antibiotics through education and training. A neonatal intensive care specialist stated, “At SBOMS, we are working to implement unified guidelines in internal medicine, ICU, and NICU [neonatal intensive care]. We have successfully piloted this approach in one NICU department, and we have plans to expand it to other NICU departments across five hospitals.” Some NGOs also provide channels with infectious disease specialists abroad who volunteer to support physicians in Syria, but this is reported as a small-scale initiative with the ability to support only a few cases.

Two participants reported that an AMS program was implemented by two different international NGOs. This included restrictions to antibiotics prescriptions according to guidelines with a system to monitor each prescriber’s behavior by monthly reports. A physician explained, “We have a WHO system in place, which is AWaRe. At the end of each month, each physician will see how much he prescribed from each category, and he should justify any increase in Watch prescription.” Another doctor who is undertaking a residency training program with SBOMS reports that they are held to account through careful monitoring of prescriptions which they must justify based on available guidelines.

A participant noted: “In our pilot project, I was able to reduce the inappropriate prescription of antibiotics in in-patient children admitted for gastrointestinal infection from 100% to around 25–30%. I headed this pilot, and I reviewed every patient’s file, to check if there’s an indication to prescribe antibiotics and discuss it with the prescriber, so every prescriber needs to have a justification for using the antibiotics, sometimes, an article, a study, or another source of information.”

#### 3.4.2 Diagnostics

Almost all participants noted challenges related to the availability and quality of microbiology diagnostics in northwest Syria which impacted AMS. They reported that without strengthening microbiology laboratories across different regions, prescribing would continue to be affected as health workers would be concerned about AMR. A paediatrician stated: “Securing sensitivity and culture tests is essential, and this is the first thing that we should start with. Even when we approached those specialists abroad, they say, ‘without culture tests do not talk to me, I cannot give you any advice’.” Some asked for more advanced testing, e.g., molecular testing as a means of bypassing some of the challenges in accessing basic microbiology services. A physician said: “Providing more advanced tools for diagnosis, will minimize the use of empirical treatment with antibiotics, and will give us better insight to treat patients.”

#### 3.4.3 Training and accountability of health workers

Most of the participants highlighted the need for the training of healthcare providers as an important recommendation to tackling AMR, including for pharmacists who were often a first line for patients to access antibiotics. Participants emphasized that the training should be also customized to the settings in northwest Syria in order to be implementable and appropriate. A paediatrician mentioned: “We need training including around the best antibiotics to use, which should be combined with practical case discussion sessions and show an understanding of our circumstances in the region. We had similar training for infant resuscitation, and it had a great impact!.”

Many participants also noted the necessity of an integrated training within a more advanced program. This advanced program would involve setting and customizing guidelines to suit the current context, training physicians based on these guidelines, and subsequently executing, monitoring, and evaluating both the adherence and the impact of the program. Key informants mentioned similar programs conducted in the region before, such as the Community Management of Acute Malnutrition “CMAM” for malnourished children and non-communicable diseases “NCD” programs. They believe these programs were successfully implemented, and physicians are strictly adhering to the guidelines provided. A paediatrician said: “We need to replicate the success of the CMAM program, a program which has been active for a few years. Now all physicians are following the program guidelines, even when prescribing antibiotics for malnourished children.” They also discussed that this approach has the potential to make physicians accountable for their prescriptions through proper monitoring.

#### 3.4.4 Policy level recommendations

All participants stressed the need for laws and policies which regulate the use of antimicrobials, by eliminating over-the-counter dispensing by pharmacies. Such a law was already in place and mostly enforced according to participants for other drugs in the region like anaesthetics and antipsychotics which are restricted; fines and revoking of licenses were often enforced if there was non-compliance. Some participants noted that implementing such a law could be challenging due to the community’s poor financial situation (which pre-empts them accessing a private physician rather than a pharmacist first) alongside the inability to absorb all patients into the public sector health system.

A pharmacist said: “The ideal solution is to have a law which prohibits dispensing antibiotics without a prescription…; when an inspector comes from the syndicate of pharmacists, they ask to see the dated and stamped prescription for every dispensed anaesthetic or antipsychotic’ medication.” A paediatrician described his attitude toward implementing such a law: “This will be a huge step, but I think it’s very difficult to be adopted by the decision makers; even if they issue it, they may not be able to implement it as there’s a huge implementation hardship.”

Another recommendation was to develop and implement policies within the framework of current community health programs. The objective of these new policies is to integrate AMR messaging and awareness topics into the existing subjects that community health workers provide information on, which can be done by either the same teams or dedicated campaigns. The community health program was reported to have good camp coverage, which is considered the most critical area to focus on. A participant stated, “They are visiting every house and tent, we should add awareness messages and promotion about AMR to their programs.” Another stated: “There should be dedicated mobile teams to raise awareness about AMR, as they did with COVID there should be posters about AMR, people should know that antibiotics are not always a good thing!”

Some participants reported the need to have a collective information system to monitor the main trends of resistance infection, as part of a surveillance system but that this would require stronger laboratory services. On a hospital level, most of the participants highlighted the needs to improve the implementation and monitoring of the IPC programs. Key informants emphasised the multiple challenges to implement IPC protocols, including the absence of dedicated staff, poor program leadership on the level of hospitals and departments, lack of resources and lack of proper infrastructure. A neonatal intensive care specialist said: “We have two things that we cannot put apart at all; [these are] following the antibiotics guidelines and implementation of IPC. Even if we have a proper usage of antibiotic guidelines, if we do not have a proper IPC the infant will be infected.”

## 4 Discussion

To the best of our knowledge, this exploratory study was the first study which explored the perceptions of healthcare professionals on AMR in former northwest Syria. Our findings suggested that there was good knowledge around AMR and a strength of feeling among participants it was an urgent matter which needed to be addressed; there was also a good understanding of the local drivers for the development of AMR and what interventions were needed. In contrast, knowledge around AMS was more limited though some of the components, e.g., antimicrobial protocols, restricted use of certain antibiotics in certain facilities were occasionally mentioned. There was more awareness of IPC though the degree to which IPC interventions were successfully implemented was reported to be variable.

An array of drivers relevant to the local context were noted which include recognized drivers of AMR as well as contextual factors such as unrestricted over the counter antibiotic availability and unregulated antibiotic prescribing, particularly among mobile health clinics., In terms of practical recommendations, some areas of good practice were highlighted which could be expanded; however, participants emphasized the need for policies and regulations which restrict antibiotics. At the time of the study (before the fall of the regime,) meaningful introduction and enforcement of such policies would not have been possible, however, now that this area falls under the new Syrian government, such interventions may be possible. Though this research was designed and interviews conducted before the fall of the Syrian regime on 8^th^ December 2024, we suggest some of our findings remain relevant to the former northwest Syria with others generalizable to wider geographies in Syria.

### 4.1 An urgency to act on AMR

During the interviews, we noted more limited mention of the term of AMS though participants were aware of the principles; where stewardship programs existed, they were reported poorly enforced and limited in sustainability. AMS programs provide an essential, immediate and cost-effective intervention which could alter prescribing practices and influence AMR. Though they are an essential pillar of the Global Action Plan to combat AMR (21) with good evidence on their impact (22), data from conflict affected settings are more limited. In Yemen, a country which faces similar challenges related to AMR and conflict, Orubo *et el* described poor AMS across the country with poor availability of prescribing guidelines or the presence of drugs and therapeutics committees (23). However, in Jordan, MSF reported the successful implementation of an AMS program in their war-surgery hospital in Amman where they treat both Syrian and non-Syrian patients. Through their program, they were able to reduce broad spectrum antibiotic prescribing and associated costs by 37% in 1 year (24).

The importance of engagement with pharmacists and patients on AMS has long been recognized as an important strategy for the success of such programs; however, our research finds limited mention of engagement. Lai et al. describe pharmacists as having an important role as ‘guardians of antimicrobials’ and highlighted the need to empower pharmacists and support their work in AMS programs, enforcement of prescribing policies and the routine monitoring antibiotic prescriptions (25). Such measures must be considered in Syria though power discrepancies between physicians and pharmacists need to be addressed. However, pharmacists, through their role in running private pharmacies and dispensing prescriptions in Syria are important to engage with.

This research provided important insights into the contextual drivers of AMR in northwest Syria, a relatively unique setting at the time of the interviews. The drivers of AMR have been widely described in the literature; however, participants reported these to be amplified in the setting of conflict. Participants named “poor general conditions” as the driver of AMR; by this they inferred the complex array of factors in conflict settings such as northwest Syria including the fragmented health system, economic collapse, poor laboratory infrastructure and the general living conditions which include overcrowding and environmental factors (11). Abbara et al. captures some of these in their review of conflict related drivers of AMR across Syria (11); additional factors highlighted by our research include the role of patients and pharmacists in prescribing practices which influence AMR and the negative impacts on antibiotic overuse or misuse for example, in mobile medical clinics. The latter are needed given the number of people living in camps, the poor transport links and the general poor living conditions which they face, however, tracking of prescribing and accountability are important to avoid inappropriate prescribing. Community engagement and education, alongside other measures are essential components of addressing this (26).

### 4.2 Contextualized approaches based on this research

The data from this research provided an opportunity to support early and longer-term recommendations appropriate to the context. Some of the factors noted in this research in former northwest Syria may be generalisable across the whole of Syria whereas, given the nature of the local context and health system, others may be less relevant. A gap noted by many of the KIs related to inadequate awareness around AMR among the public, something which is important (21), particularly in northwest Syria. Though free healthcare provided by humanitarian organizations predominated in this area (unlike in other areas of Syria), patients may still use their agency to purchase over the counter antibiotics should they not receive them directly, when they request them. This could be addressed through strengthening community engagement initiatives and health promotion programs which both international and local humanitarian organizations have been doing, particularly in the wake of the COVID-19 pandemic (27). This can be done through empowering community health workers, something which has seen some success in other community initiatives including COVID-19 vaccination uptake (28). A report by Relief Experts Association, an implementer of community health programs noted that they could achieve high coverage in their target locations with good adherence to introduced standard operation procedures (29). Research from other settings, stresses the importance of addressing community level and behavior change interventions in low resource settings to tackle AMR at the local level. Such approaches may require tailored approaches to different groups or sub-groups in the region given the differential impacts on age, gender, education level or living conditions as examples (16).

The need for strengthening and implementing sustainable interventions for IPC were noted but most participants given the risks associated with nosocomial spread of MDROs. Though there have been various programs introduced by both WHO and humanitarian organizations according to WHO standards, there remains a gap on the ground in effective engagement with IPC measures and monitoring despite the training of more than 500 health workers in the area (27). Evidence from a pooled systematic review showed the IPC interventions can reduce healthcare associated infections by 30–70% (30). Conflict settings provide challenges to the implementation of standard IPC protocols due to poor planning or buildings, e.g., converted houses or schools that were not purpose built as hospitals, a lack of space and poor provision for screening and isolation measures. El Mouallem et al. describe some of these challenges in Syria and other conflict settings including Yemen and Gaza (31). This review and our KIs stressed the importance of leadership and accountability to ensure appropriate IPC interventions.

Despite good standards of knowledge around AMR displayed by the KIs, there remained a need for training of physicians, pharmacists and other healthcare workers, particularly community healthcare workers, nurses, and technicians given their important roles in the health system. Research from other low resource settings also highlights the discrepancies in knowledge on AMR among different health cadres (32). However, education and training alone are not effective. In research by Esmaily et el (33), and Shrestha et el (34), from Iran and Nepal, both of which have been affected by armed conflict, found only partial or no improvement in prescribing behaviors if it were a stand-alone intervention. However, multi-faceted educational interventions have shown significant improvement (35). Cuevas et al.’s systemic review presented evidence of effective programs, particularly where theoretical education and practical training can be effective when combined with “feedback and mentorship program” (36). This was corroborated by Afari-Asiedu et al.’s systematic review (37).

In the longer term, systems strengthening approaches focused on supporting laboratory and diagnostics, IPC and AMS with embedded programs of education for undergraduate and postgraduate healthcare workers across Syria are important. This must occur alongside the provision of required consumables, e.g., disinfectants, sterilisation materials and sustained antibiotics supplies to avoid ruptures. Actional measures include policies which restrict over the counter dispensing of antibiotics without prescriptions, accountability and monitoring of antibiotics prescribing in both community and inpatient settings and a focus on the WHO AWaRE antibiotics. Such laws existed in Syria before the conflict however, they were not enforced. In the current situation across Syria, particularly in rural areas or those poorly served by healthcare, pragmatic approaches to restrictions should be taken as over-restriction could lead to poor outcomes for vulnerable patients in these settings (41).

Though Syria launched a National Action Plan on AMR in 2019, there was no implementation, and the former northwest of Syria had been excluded as it did not fall under the former Ministry of Health. With the new Syrian Ministry of Health, there are opportunities to revise and update the National Action Plan on AMR in a way suitable to the new context and with clear pathways to implementation. This should include multilateral rather than vertical programs as found by reviews from Shen et al. (38), and Wilkinson et al. (39, 40).

### 4.3 Strengths and limitations

To our knowledge, this is the first research which qualitatively explored the perspectives of healthcare workers on AMR in Syria. Through this, we highlighted some of the contextualized factors which have previously been underexplored. Limitations of this study include that this study is focused on the former northwest of Syria, an area which now falls under the new Damascus based Ministry of Health. This dramatic change in the circumstances mean that though some of our findings remain relevant, some may not be generalisable to the rest of Syria, given the unique factors which led to the evolution of the health system in each of these areas. Another limitation is that our study focused on the perspectives of pharmacists and physicians whereas other healthcare providers, e.g., nurses, community health workers, dentists may also influence prescribing practices and community perspectives. Further research which explores their perspectives and the perspectives of the community are needed with an exploration of what future recommendations are practicable in the current situation in Syria.

## 5 Conclusion

Tackling AMR in Syria requires multifaceted approaches during the current, early recovery phase of the health system. Understanding the perspectives of healthcare workers in the former northwest Syria around AMR, can provide useful insights into what can be done to tackle this systemic issue. There are some factors noted by the participants which are unique to the context in the former northwest Syria while others may be more generalisable to the rest of Syria. As such, recommendations which recognize these similarities and differences are needed to ensure that contextually appropriate recommendations are made.

## Data Availability

The raw data supporting the conclusions of this article will be made available by the authors, upon reasonable request.

## Appendix. Interview Guide

Personal Information:

**Table:**

Key informant code:	Gender:	Age:
Current occupation:	Education Level:	Place of residence:

- Have you worked in Idleb governorate in a clinical capacity during the last 5 years?
- Are you involved in the care of patients with infections which require antibiotics in the course of your work?
- Have you been working in Idlib governorate around the time of the earthquake? Please expand.

KAP on extent, drivers, guidelines and management.

1. What do you understand by the term AMR? What do you understand by the term AMS?
2. What do you know about the extent of AMR in northwest Syria? How important it is?
3. Tell me about any existing guidelines on antibiotic prescribing, antibiotic stewardship, management of infection or similar in northwest Syria? How effective are these guidelines? If not very effective, what changes could make them more effective?
4. What do you think are the contributors to AMR in northwest Syria?
5. Can you walk me through your approach to handle a suspected AMR case?

The effect of the protracted armed conflict forced displacement and earthquake.

1. In what way do you think that the armed conflict could impact AMR in northwest Syria?
2. In what way do you think that the conflict related forced displacement could impact AMR in northwest Syria?
3. What effect do you believe the earthquake could have on AMR in northwest Syria?

Best practices and recommendations.

1. What examples of interventions to address AMR in northwest Syria are you aware of? How effective are they?
2. What recommendation do you have as to how to limit the extent of AMR in northwest Syria?
